# The Mechanism of Downregulated Interstitial Fluid Drainage Following Neuronal Excitation

**DOI:** 10.14336/AD.2020.0224

**Published:** 2020-12-01

**Authors:** Yuanyuan Li, Hongbin Han, Kuangyu Shi, Dehua Cui, Jun Yang, Ian Leigh Alberts, Lan Yuan, Guomei Zhao, Rui Wang, Xianjie Cai, Ze Teng

**Affiliations:** ^1^Department of Radiology, Peking University Third Hospital, Beijing, China.; ^2^Beijing Key Laboratory of Magnetic Resonance Imaging Equipment and Technique, Beijing, China.; ^3^Institute of Medical Technology, Peking University Health Science Center, Beijing, China.; ^4^Department of Nuclear Medicine, University of Bern, 3010 Bern, Switzerland.; ^5^Department of Informatics, Technical University of Munich, Garching 85748, Germany.; ^6^Peking University Medical and Health Analysis Center, Peking University Health Science Center, Beijing, China.; ^7^Department of Radiology, Cancer Hospital Chinese Academy of Medical Sciences, Beijing, China.

**Keywords:** neuronal excitation, brain extracellular space, interstitial fluid, tracer-based MRI, neurotransmitters

## Abstract

The drainage of brain interstitial fluid (ISF) has been observed to slow down following neuronal excitation, although the mechanism underlying this phenomenon is yet to be elucidated. In searching for the changes in the brain extracellular space (ECS) induced by electrical pain stimuli in the rat thalamus, significantly decreased effective diffusion coefficient (D_ECS_) and volume fraction (α) of the brain ECS were shown, accompanied by the slowdown of ISF drainage. The morphological basis for structural changes in the brain ECS was local spatial deformation of astrocyte foot processes following neuronal excitation. We further studied aquaporin-4 gene (*APQ4*) knockout rats in which the changes of the brain ECS structure were reversed and found that the slowed D_ECS_ and ISF drainage persisted, confirming that the down-regulation of ISF drainage following neuronal excitation was mainly attributable to the release of neurotransmitters rather than to structural changes of the brain ECS. Meanwhile, the dynamic changes in the D_ECS_ were synchronized with the release and elimination processes of neurotransmitters following neuronal excitation. In conclusion, the downregulation of ISF drainage following neuronal excitation was found to be caused by the restricted diffusion in the brain ECS, and D_ECS_ mapping may be used to track the neuronal activity in the deep brain.

The brain extracellular space (ECS) is a direct microscopic environment in which brain cells survive and function; it occupies 15-20% of the whole brain volume [1]. Under a microenvironment of absence of lymphatic vessels in the brain parenchyma, interstitial fluid (ISF) within the brain ECS is directly exposed to the extracellular matrix and cellular membrane of various brain cells [1-4]. Both cellular and vascular compartments are reportedly in the production and metabolism of ISF. The transportation of substances in the brain ECS is considered to play critical roles in memory, sleep and perception, and is implicated in the pathophysiology of neurodegenerative disorders, such as Alzheimer's disease [5-8].

Previous studies have shown that the transportation of substances within the ECS can be regulated by endogenous aquaporins on astrocytes, the integrity of myelin, and external neuronal stimulation [9-11]. Oligodendrocytes extended their processes wrapping around neuronal axons to facilitate the bio-electrical conduction between neurons, and interestingly, a new function of myelin has been revealed as ramping, guiding, and separating the ISF drainage of the brain [9]. Meanwhile, it has been reported that aquaporin-4 gene (*AQP4*) deficiency impairs the ISF drainage in the brain [10, 12]. Recent findings have indicated that neuronal activities following painful and olfactory stimuli slow down the ISF drainage in the ECS division where the excitatory nucleus is located [11, 13]. Functional electrical stimulation displays unparalleled superior characteristics and has unique efficacy in the treatment of and body-function recovery form neurological and psychiatric diseases (such as epilepsy, depression, and Parkinson’s disease) [14]. However, the dynamic modulatory processes that occur in the ECS upon stimulation, as well as their underlying mechanisms, have not been elucidated.

**Figure 1.**
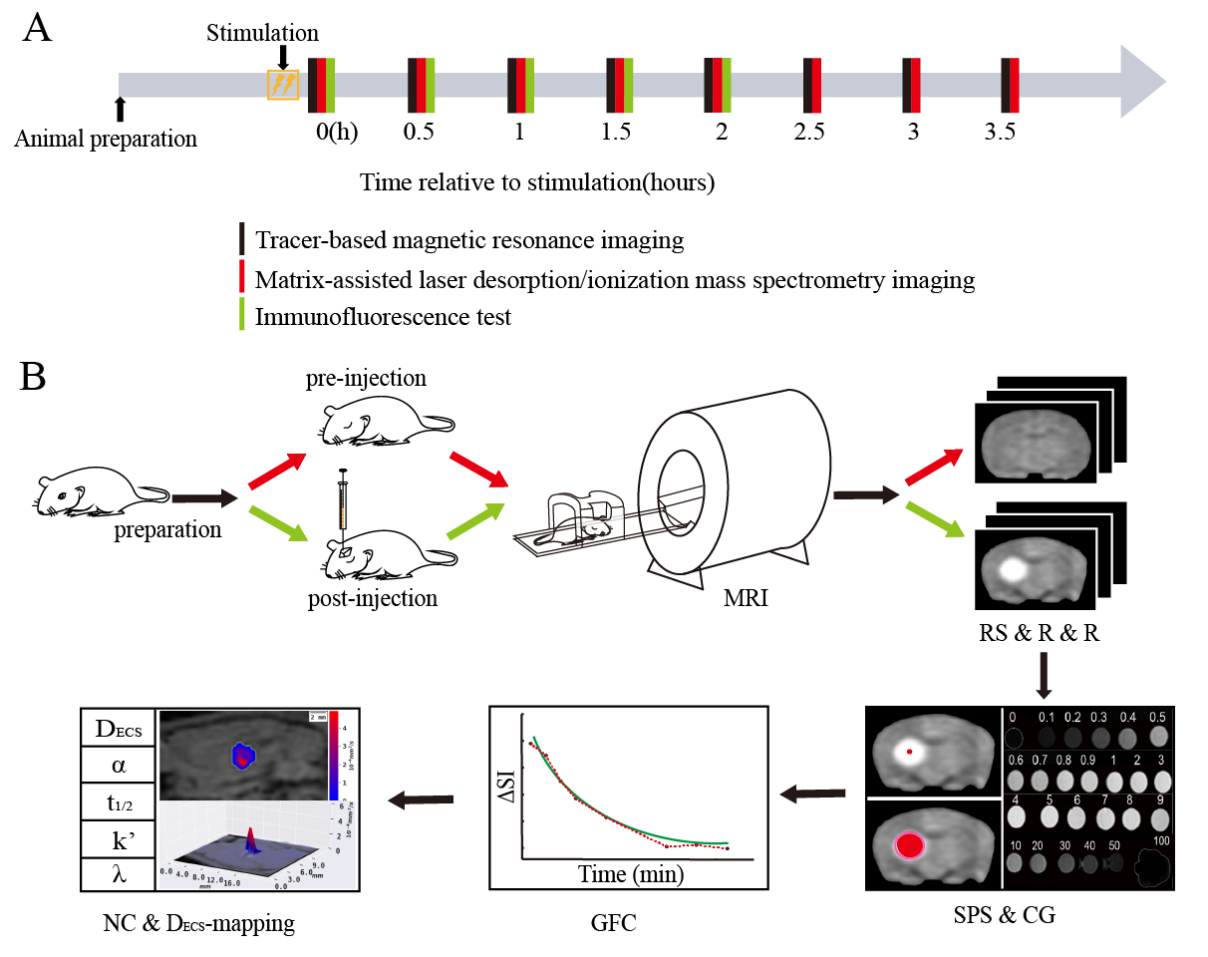
Flowchart of the experimental procedure. (A) Time course of the experiment. Tracer-based MRI scanning (black line), MSI (red line), and immunofluorescence assays (green line) were performed on the rats at different timepoints after electrical forepaw stimulation (0, 0.5, 1, 1.5, 2, 2.5, 3, and 3.5 h). (B) Experimental flowchart of tracer-based MRI. MR scanning was performed on the experimental rats under anesthesia before and at different timepoints after tracer injection until the high-intensity signals disappeared in the MR images. The enhanced local brain region of each rat at each timepoint was obtained by three-dimensional registration and subtraction of the MR signals from the images before tracer injection. The seed point in the region with tracer signal on the rat brain image acquired at the initial timepoint after tracer injection was selected as the starting point for diffusion. Subsequently, the images were processed using software developed in our laboratory. Finally, the diffusion coefficients of the microstructure in the brain ECS was obtained. Abbreviations: ECS: extracellular space; MRI: magnetic resonance imaging; MSI: mass spectrometry imaging; RS & R & R: region segmentation and registration and reduction; SPS & CG: seed point selection and computational growth; GFC: generation of fitting curves; NC & D_ECS_-mapping: numerical calculation and D_ECS_-mapping.

Given the potentially important capacity of tracer-based magnetic resonance imaging (MRI) in visualizing the drainage of ISF in the brain ECS and analyzing the diffusion characteristics of the ECS in the deep brain [15, 16], it is pertinent to explore the underlying biophysical mechanisms. In the present study, using tracer-based MRI and effective diffusion coefficient (D_ECS_)-mapping techniques, we quantitatively measured the dynamic biophysical parameters of the brain ECS structure and ISF drainage following neuronal excitation in the thalamus of electric pain stimulation rat model. Immunofluorescence assays and western blot analysis were used to confirm the morphological basis for structural changes in the ECS. Additionally, the release and distribution of neuro-transmitters were mapped at different timepoints following pain stimuli by using mass spectrometry imaging (MSI). The same examinations were performed in an *AQP4* knockout rat model to explore the roles of *AQP4* in regulating ECS structure and ISF drainage. These data should allow us to delineate the mechanism by which neural networks interact with the ECS following neuronal excitation, providing new insights to elucidate the processing of perceptual information in the brain.

## MATERIALS AND METHODS

### Animal preparation

The animal experimental protocols were approved by the Experimental Animal Welfare and Ethics Board of the Peking University Health Science Center Biomedical Ethics Committee. We used adult male Sprague Dawley rats that weighed between 250-300 g. Rats were randomly assigned to the control group (CG; *n* = 6), the stimulation group (SG; various timepoints relative to electrical stimulation: 0, 0.5, 1, 1.5, 2, 2.5, 3, and 3.5 hours; *n* = 6 per group) (Fig. 1A), the aquaporin-4-deficient group (*AQP4*^-^; *n* = 3), or the stimulation group with aquaporin-4 deficiency (SG-*AQP4*^-^; *n* = 3). MRI scan and immunofluorescence assay were performed at the 0 h timepoint after electrical stimulation, and MSI was performed at 2 h after electrical stimulation in the SG- *AQP4*^-^ group. The *AQP4* knockout rats were prepared using transcription activator-like effector nuclease-mediated knockout with a method previously described, and the effectiveness of the knockout was validated using gene detection and protein quantification [10]. Rats were housed in an enclosed breeding room with a time-controlled lighting device providing the appropriate day-night cycles (12 h light, 12 h dark). The breeding room’s temperature was maintained at 20-25°C, with relative humidity at 50-65%. All experimental animals were subjected to the same anesthesia requirements for standardization. Rats were anesthetized via intra-peritoneal injection of a combination of anesthetics (including sodium pentobarbital, ethanol, chloral hydrate, magnesium sulfate, and propylene glycol) at a dose of 0.3 ml/kg bodyweight. Afterwards, the rats were monitored every hour and administered an appropriate amount (0.1-0.3 ml) of additional anesthetics based on their condition. Following anesthesia, the apparatus for physiological measurements was used to monitor the heart rate, respiratory rate, mean arterial pressure, and partial pressure of oxygen (PaO_2_) of the rats. The body temperature of the rats was maintained at 37-38°C throughout the experiment.

### Rat electrical pain stimulation model

In the electrical pain stimulation model, the innervation territories of the median nerve under the second and fourth digits of the left forepaw of each rat were used as stimulation points. Two smooth stainless-steel needle electrodes (26 gauge) (Genuine Grass instruments, West Warwick, USA) were inserted 2-4 mm apart. The stimulation electrode covered a saline-saturated cotton ball and was fixed with tape. The excitatory responses of the thalamic neurons were induced by the current flow between the two electrodes. Stimulation was delivered through a rectangular pulse with an intensity of 3 mA and a frequency of 3 Hz using a block design activation approach: OFF-ON-OFF. The stimulation was delivered over a period of 10 min (each stimulation lasted for 15?s with a 5?s interval) [11]. Results from earlier electrophysiological examinations and analysis showed that electrical stimulation of the forepaw could lead to neuronal excitation in the contralateral thalamus [11]. The stimulated areas of the skin were intact in all rats at the completion of the experiments with no evidence of electrical burns. Electrodes were inserted under the skin of rats in the CG, but electrical stimulation was not applied.

### Intracranial tracer infusions

To ensure the accuracy of the puncture position, an MR scan was performed prior to drug administration to determine the needle trajectory and depth. An incision was made in the scalp from the interaural area to the interocular area of the rat, and the tissue over the scalp was carefully reflected to expose the bregma. The rat was immobilized in a stereotactic coordinate system (Lab Standard Stereotaxic-Single, Stoelting Co, IL, USA) in the prone position. The position for the trephine hole was located on the skull surface according to the MRI pre-scan results. A total of 2 μl soluble gadolinium-diethylene-triaminepenta-acetic acid (Gd-DTPA, 10 mmol/L, Magnevist; Bayer Schering Pharma AG, Berlin, Germany) tracer was injected into the right thalamus (3 mm posterior, 3 mm lateral, and at a depth of 6 mm relative to the bregma) at a rate of 0.2 μl/min using a microsyringe (Hamilton, Bonaduz AG, Bonaduz, Switzerland) and an automated drug administration system (Harvard Apparatus, Holliston, MA, USA). After the injection, the needle was retrieved to prevent reflux of the contrast agent along the needle passage. After the skin was sutured, rats were injected subcutaneously with antibiotics (ampicillin, 100 mg/kg) and analgesics (flunixin meglumine, 2 mg/kg) to prevent infection and relieve pain.

### Tracer-based magnetic resonance imaging

To observe the effects of electrical stimulation on substance transportation in the ECS of the thalamus, an algorithm-optimized tracer-based MRI method was applied to scan the whole head of the rats at different timepoints after electrical stimulation (0, 0.5, 1, 1.5, 2, 2.5, 3, 3.5 h) (Fig. 1A). All animals underwent MRI scanning at various timepoints (15 min, 30 min, 45 min, 1 h, 1.5 h, 2 h, 2.5 h, 3 h, 3.5 h, 4 h, 4.5 h, 5 h, 5.5 h, and 6 h) after the tracer injection until the high-intensity signals of the tracer disappeared (Fig. 1B). Scanning was performed using a 3.0 T MRI system (Magnetom Trio, Siemens, Munich, Germany) in conjunction with an 8-channel wrist surface coil with animals in the prone position [17] (Fig. 1B). MRI scanning protocols were performed as previously described to obtain the three-dimensional (3D) spatial distribution of tracer concentration in the brain parenchyma. Based on the modified diffusion equation and least squares fitting technique, the diffusion and distribution of tracer in the ECS were quantitatively processed and analyzed using the NanoDetect analysis system software, version 2.1 (MRI Lab, Beijing, China) [10, 18]. Quantitative parameters on the ECS structure and ISF drainage were also obtained including the D_ECS_, tortuosity (λ), clearance rate (k’), and volume fraction (α). Once the time and volume parameters were derived, the fitted concentration-time curve and half-life (t_1/2_) were obtained. After obtaining MR images of the rat brains, we drew 2D and 3D wireframe D_ECS_-mapping with a Python package called matplotlib (Fig. 1B). The height of the figure represents the signal intensity of the MR image. The XY plane at the bottom of the figure is a contour map superimposed on the original MR image (Fig. 1B).

### Immunofluorescence assays

To assess the morphological changes in astrocytes in the rat thalamus caused by external electrical stimulation, immunofluorescence was used to specifically label astrocytes (CG; SG-0, 0.5, 1, 1.5, and 2 h; *n* = 6 per group) (Fig. 1A). After a series of dehydration, immersion, and embedding, the decapitated rat brain was sectioned into 8-μm-thick slices. The sections were blocked with 10% goat serum and punched with 0.25% Triton-phosphate buffer saline (PBS) for 10 min. Anti-glial fibrillary acidic protein (GFAP) antibodies (1:200) were diluted to the working concentration and added to the sections until the specimens were completely covered. Primary antibodies were replaced with PBS for negative controls. After incubation, the sections were immersed and washed three times (10 min per wash) in PBS; then, the secondary antibody, CY3 goat anti-rabbit IgG (1:300), was added. After incubation, the sections were immersed and washed in PBS, and then filter paper was used to remove the excess water without drying the specimen.

To localize the cells under ultraviolet light, a drop of 4',6-diamidino-2-phenylindole (DAPI) was added to the slide to label the nuclei of the astrocytes. The slides were covered with a cover slip, sealed with a neutral resin, and observed under a laser confocal microscope. Images were acquired using an inverted fluorescence microscope (Nikon Eclipse Ti-SR, Nikon, Tokyo, Japan), and the results were analyzed using the Image-Pro Plus 6.0 (Media Cybernetics, Inc., Rockville, MD, USA) and Imaris-x64 9.2.1 software (Bitplane). The average process length (PL) and process mean diameter (PMD) of the astrocytes were measured in the immunofluorescent images. We also measured the mean optical density (MOD) of the immunofluorescence using the Image-pro plus 6.0 software (Media Cybernetics, Inc., Rockville, MD, USA).

### Western Blot (WB)

GFAP levels in the thalamus of rat brains were detected by western blot analysis (CG; SG-0 h; *n* = 4 per group). Briefly, protein lysates were obtained using radioimmunoprecipitation assay lysis buffer (Servicebio, Wuhan, Hu Bei, China), and 50 μg of each sample was loaded onto a 12% tricine-sodium dodecyl sulfate-polyacrylamide gel and subsequently transferred to a polyvinylidene difluoride membrane (Millipore, Burlington, MA, USA). Membranes were blocked for 1 h with 5% nonfat milk and incubated overnight at 4°C with primary antibodies against GFAP (rabbit anti-rat; 1:1000; Abcam) in tris-buffered saline, 0.1% Tween 20 (10 mmol/L Tris, pH 8.0, 150 mmol/L NaCl, 0.05 % Tween 20) containing 3% nonfat milk. Membranes were then incubated for 1 h at room temperature with the secondary antibody (goat anti-rabbit; 1:1000; Abcam). Proteins were detected by chemiluminescence and analyzed using the Alpha software (Alpha Software Corporation, Burlington, MA, USA). We measured the grayscale values of the control and stimulation groups, and calculated the ratios of the grayscale values of the target bands to those of the internal reference bands.

### MALDI mass spectrometry imaging

Rats were anesthetized at different timepoints following electrical stimulation (CG; SG-0, 0.5, 1, 1.5, 2, 2.5, 3, and 3.5 h; *n* = 6 per group) (Fig. 1A). The intact brain was quickly isolated after decapitation. The brain was snap-frozen in solid carbon dioxide for 20 s and rapidly transferred to a -80°C freezer for storage. The temperature of the microtome was set to -20°C during sectioning. The temperature of the object head was set to -18°C and was equilibrated for more than 3 h. The brain tissue was retrieved from the -80°C refrigerator and placed in the microtome to equilibrate with the internal environment of the microtome for 30 min or more. We adhered the brain tissue to the object head via freezing with physiological saline. At least one flat intact brain section was cut from the thickest section of the posterolateral nucleus of the thalamus, with thickness 10 μm, and transferred to an indium tin oxide-coated glass slide by thaw-mounting. The brain section was thawed using the temperature of the finger on the back of the slide so that the brain tissue could be mounted securely onto the slide.

The sections collected for MSI were placed in a vacuum drying pump for 30 min, and then the tissue sections were spray-coated with matrix using the ImagePrep matrix deposition device for MALDI imaging. A solution of 1,5-diaminonaphthalene hydrochloride had been pre-prepared by adding 500 μl of 1 mM hydrochloric acid solution, followed by 4.5 ml of distilled water to 39.5 mg of 1,5-diaminonaphthalene. The mixture was dissolved using ultrasonography until no particles were visible. High-grade absolute ethanol (2 ml) was then dissolved in the solution, and it was stored in the dark. The 1,5-diaminonaphthalene hydrochloride was placed into the spraying device. The spray intensity was tested and adjusted until the matrix solution could be evenly sprayed on the entire slide. The spray conditions were as follows: spraying, 1 s; incubation, 20 s; and drying, 70 s. The spray cycle depended on the nozzle condition, and the remaining parameters were set by default. For the second round of matrix spraying, the slides were placed onto the matrix deposition device at a 180° rotation relative to the last spray. The thickness of the matrix that adhered to the slide was observed after two rounds of matrix spraying. Matrix spraying was continued when the coating was not sufficiently thick; otherwise, the mass spectrometry procedure was executed as described below.

Prior to MSI, optical images of the slides containing the brain sections were obtained with a scanner. The imaging area was defined simultaneously at the interface of the optical images and mass spectrometry images. This experiment used the Ultraflextreme MALDI- time-of-flight (TOF) / TOF mass spectrometer (Bruker, Billerica, MA, USA), with a laser source of Nd: YAG and a wavelength of 355 nm. Data were acquired in negative-ion scan mode where the charge-to-mass ratio range was 80-1000 and the spatial resolution was 200 μm. A total of 150 shots were accumulated per image. The laser intensity was set to 50%. After the mass spectrometry was completed, the data were processed using the FLEXIMAGE software (Bruker Daltonik GmbH, Bremen, Germany). Specific charge-to-mass ratio peaks were selected to observe the distribution of various metabolism-related small molecules in the sections. The matching of charge-to-mass ratios with the corresponding small molecules was based on protocols from relevant previously published literature [19].

### Statistical analysis

All data were expressed as the mean ± standard deviation (x¯ ± SD). Both linear fitting and statistical analyses were performed using SPSS 20.0 (IBM SPSS, Armonk, NY, USA). Intergroup comparisons were performed using one-way ANOVAs or t-tests (a nonparametric test was used if data were not normally distributed). The linear relationships of different curves were quantitatively analyzed using Pearson’s correlation coefficient test. All statistical tests were bilateral, and P < 0.05 was considered statistically significant.

## RESULTS

### No significant difference in the physiological parameters between groups

All measured physiological parameters of the rats maintained within the normal physiological ranges during the entire experiment. The physiological parameters of the rats presented no statistically significant difference in these groups before and after stimulation (heart rate: 353.02 ± 6.19 bpm / 355.89 ± 10.59 bpm; respiratory rate: 53.09 ± 0.90 bpm / 53.32 ± 0.99 bpm; mean arterial pressure: 126.53 ± 5.27 mmHg / 129.16 ± 4.06 mmHg; PaO_2_: 88.02 ± 1.97% / 89.12 ± 2.89%: before / after stimulation respectively) with no statistically significant difference between these groups (P > 0.05). During stimulation, the maximum increase in systemic arterial blood pressure was approximately 5 mmHg. Signs of nystagmus, masticatory muscle movement, salivation, or tear secretion after electrical stimulation or the pulsed magnetic fields did not differ between these groups of rats.

**Figure 2.**
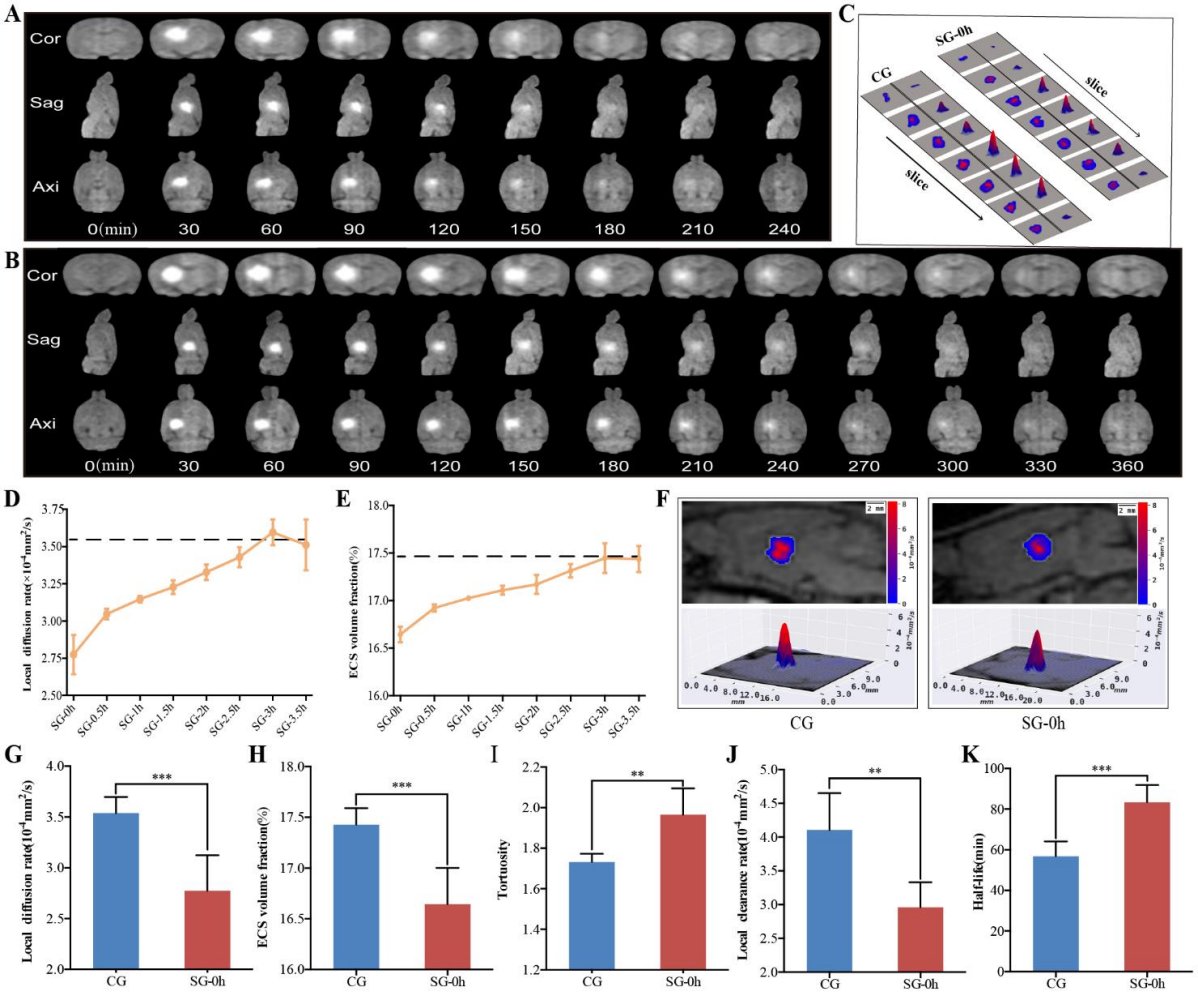
Dynamic changes in the diffusion parameters of the ECS in the thalamus induced by electrical stimulation. (A-B) Representative images showing ISF drainage using tracer-based MRI in the coronal, axial, and sagittal planes of the thalamus; the images represent six independent experiments conducted on six rats. Compared with the CG (A), the effects of the electrical stimulation were persistent and caused a delay in signal attenuation (B). (D-E) Dynamic changes in the ECS diffusion parameters, D_ECS_, and α, in the thalamus were obtained at different timepoints after electrical stimulation (0, 0.5, 1, 1.5, 2, 2.5, 3, and 3.5 h). Compared with the CG (dashed line), changes in the D_ECS_ and α were the most significant at 0 h after stimulation. The duration of the parameter changes was longer than 2 h. At 3 h after stimulation, the ECS diffusion parameters and structural parameters of the thalamus were essentially restored. (C, F) Representative images showing the 2D and 3D D_ECS_-mapping images of the different groups. The contour maps of the D_ECS_-mapping images show that electrical stimulation causes a change in the patterns and amplitudes of the contour maps of D_ECS_ as compared with the CG. (G-K) Compared with the CG without stimuli, pain stimulation caused a marked change in the ECS structure and ISF drainage of the thalamus. The D_ECS_ and α in the SG-0h were significantly decreased compared to those in the CG. The t_1/2_ in the SG-0h was significantly higher than that in the CG. Additionally, the λ was increased and the k’ was decreased in the SG-0h when compared with those of the CG. Data are expressed as the mean ± standard deviation. ** Denotes a significant difference between the samples (P < 0.01), and *** denotes a highly significant difference between the samples (P < 0.001). Abbreviations: ECS: extracellular space; ISF: interstitial fluid; MRI: magnetic resonance imaging; CG: control group; SG: stimulation group; D_ECS_: effective diffusion coefficient of the ECS; α: volume fraction; λ: tortuosity; k’: clearance rate; t_1/2_: half-life.

### Dynamic changes in the ECS structure and ISF drainage in thalamus after pain stimulation

Our modified T1 magnetization-prepared rapid gradient-echo scanning protocol allowed us to dynamically observe the ISF drainage of rats on the whole-brain scale, which increased the sensitivity to the changes in diffusion parameters in the brain ECS induced by the stimulus. Due to the longitudinal relaxation effect of the probe, T1 images showed a signal-enhanced region in the thalamus 15 min after tracer injection and the signal-enhanced region was gradually expanded over time, while the signal intensity gradually decreased (Fig. 2A). Compared to the CG (240 min), the effects of the electrical stimulation were persistent and caused a delay in signal attenuation (360 min) (Fig. 2B). Previous studies have shown that despite being adjacent to the inner capsule, a barrier to ISF drainage is formed by the compact fiber bundling in the inner capsule region that blocks the ISF flow from the thalamus into the caudate nucleus in the brain ECS. The distribution and clearance path of ISF in the thalamus observed in this study were consistent with the theory of brain ECS divisions [1, 20].

**Figure 3.**
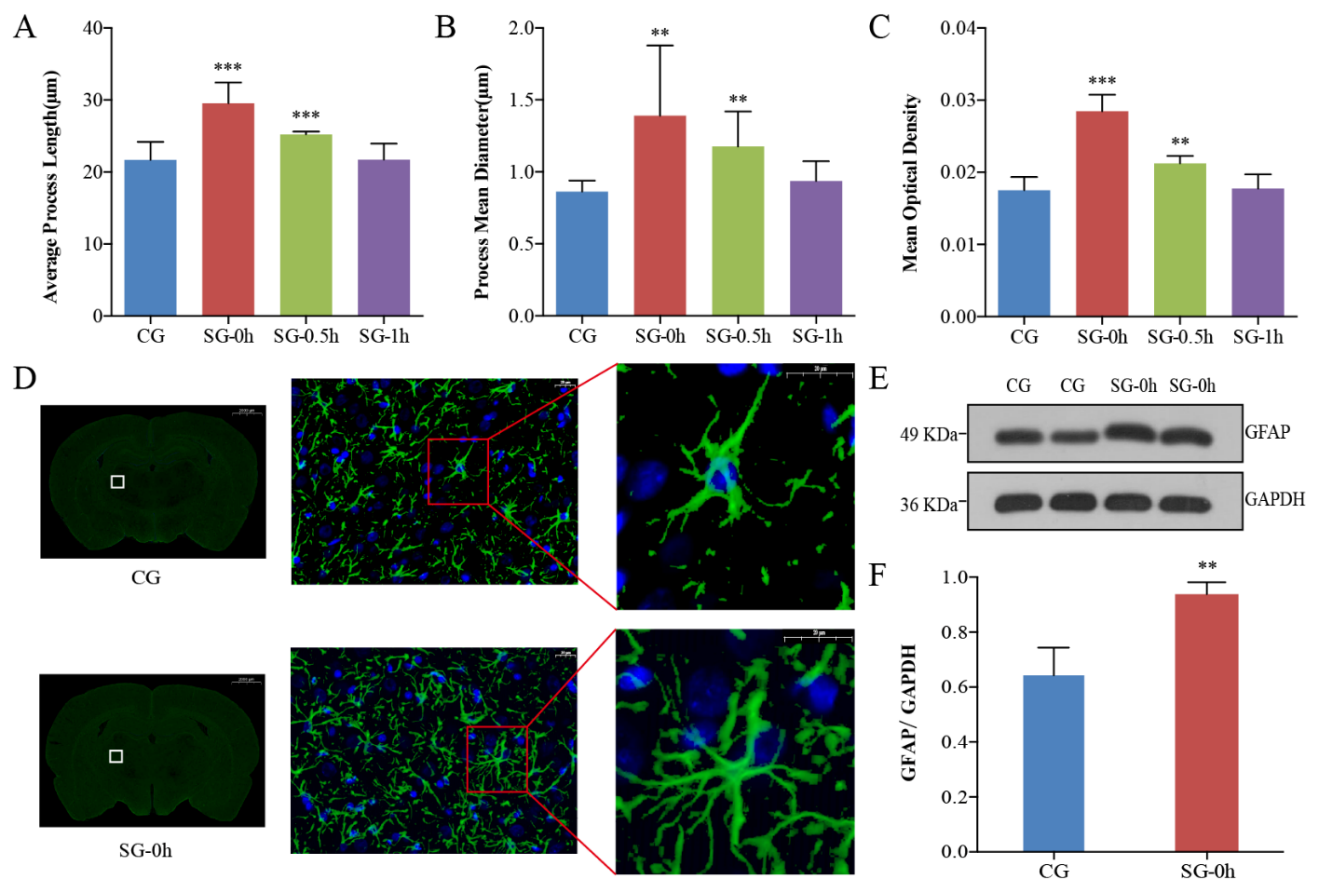
Morphological and functional changes in astrocytes in the thalamus induced by electrical stimulation. (A-B) Astrocytes were specifically labelled with immunofluorescence at different timepoints after electrical stimulation. Compared with the CG, the PL and PMD of the astrocytes were most significant at 0 h after stimulation, and the morphological changes in astrocytes were restored at 1 h after stimulation. (C-F) Compared with the CG, electrical stimulation caused the processes of the astrocytes to become thickened, extended, dense, and interlaced (D). Immuno-positive astrocytes showed enhanced GFAP immunoreactivity, with upregulated MOD and increased expression of GFAP following neuronal excitation (C, E, F). Data are expressed as the mean ± standard deviation. ** P < 0.01, *** P < 0.001. Abbreviations: CG: control group; SG: stimulation group; PL: process length; PMD: process mean diameter; MOD: mean optical density; GFAP: glial fibrillary acidic protein.

To observe the effects of electrical stimulation on substance transportation in the ECS of the thalamus, both algorithm-optimized tracer-based MRI and *in vivo* D_ECS_-mapping were applied to scan the whole head of rats at different timepoints after electrical stimulation (0, 0.5, 1, 1.5, 2, 2.5, 3, 3.5 h) (Fig. 2). This enabled monitoring of the dynamic changes in the diffusion parameters of the brain ECS in the rats. The parameters of ECS structure and ISF drainage were obtained in the CG, including D_ECS_ = (3.54 ± 0.16) × 10^-4^ mm^2^/s, α = (17.43 ± 0.16) %, λ = 1.73 ± 0.04, k’ = (4.10 ± 0.55) × 10^-4^mm^2^/s and t_1/2_ = (56.79 ± 7.37) min. Compared with the CG, pain stimulation caused marked dynamic changes in the D_ECS_ and α of the brain ECS (Fig. 2D-E). In summary, the greatest changes in these parameters (D_ECS_ and α) were observed at 0 h after stimulation (SG-0h, i.e., immediately after stimulation) (P < 0.001); then, the duration of the parameter changes was more than 2 h (SG-2h, P < 0.05); finally, the affected diffusion and structural parameters in the thalamus were largely restored at 2.5 h post-stimulation (SG-2.5h, P > 0.05) (Fig. 2D-E). Specifically, compared to the CG, the D_ECS_ and α in the SG-0h were significantly decreased (P < 0.001) (Fig. 2C, F-H). In contrast, the t_1/2_ of SG-0h was significantly longer than that of CG (P < 0.001) (Fig. 2K). Additionally, the λ was increased and the k’ was decreased in the SG-0h compared with that of the CG (P < 0.01) (Fig. 2I-J). The acquired parameters were highly reproducible both temporally and spatially.

**Figure 4.**
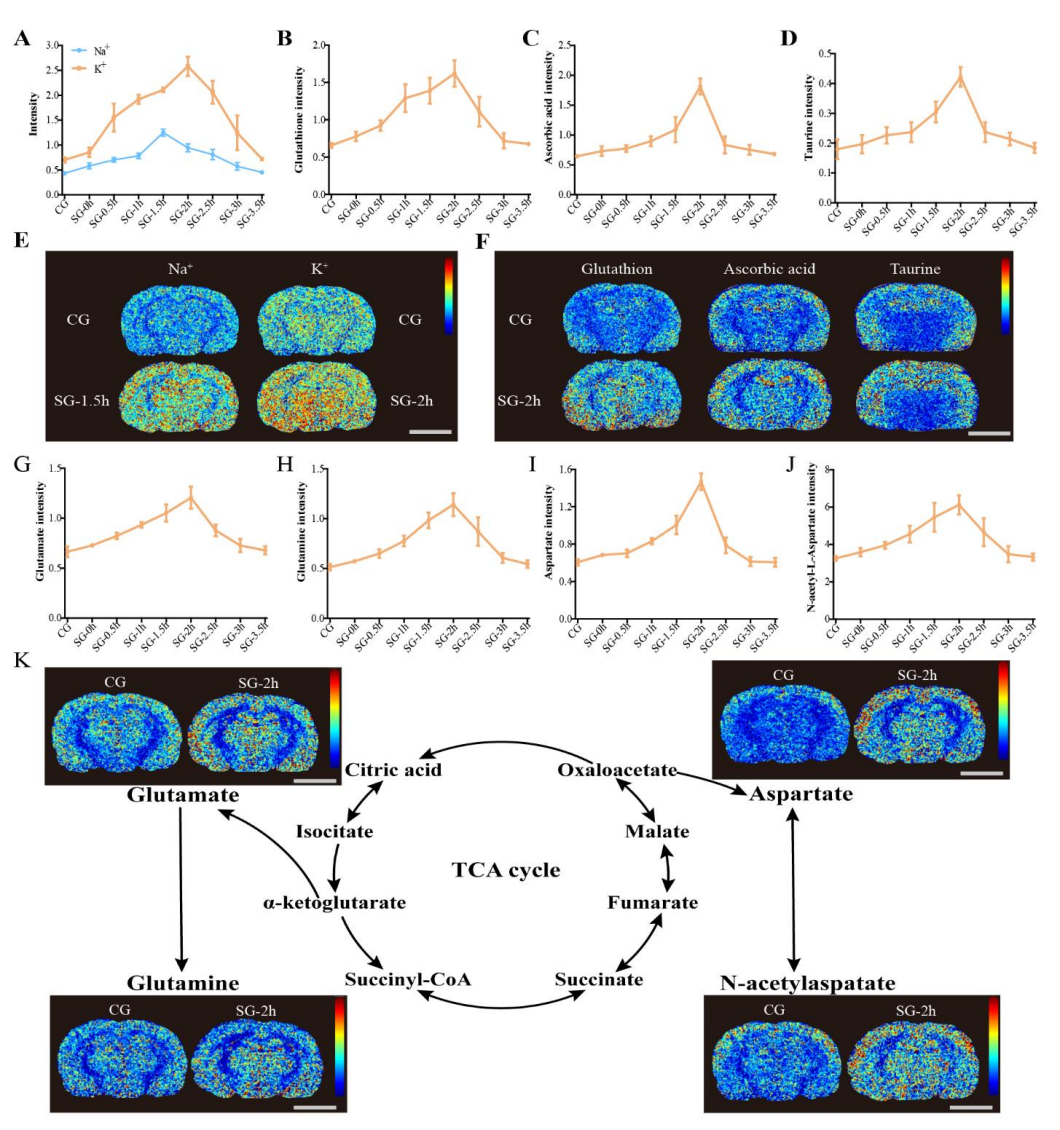
Dynamic changes in the metabolism of substances in the ECS of the thalamus induced by electrical stimulation. (A-D, G-J) Compared with the CG, electrical stimulation caused dynamic changes in the metabolism of many molecular substances in the rat thalamus, including inorganic ions, antioxidants (glutathione, ascorbic acid, and taurine), glutamate, glutamine, aspartate, and N-acetylaspartate. The most significant changes in the amount of these macromolecules were observed in the SG-2h group. The stimulation-induced metabolic changes were effectively restored after 3 h. (E-F, K) The results from the MSI images showed that the average density of metabolites was significantly increased after electrical stimulation compared with the average density of metabolites in the CG (scale bar, 5 mm). Data are expressed as the mean ± standard deviation. Abbreviations: ECS: extracellular space; CG: control group; SG: stimulation group; TCA cycle: tricarboxylic acid cycle; MSI: mass spectrometry imaging.

### Morphological and expression changes of the astrocytes after pain stimulation

To evaluate the morphological changes of the astrocytes in the rat thalamus, immunofluorescence was used to specifically label astrocytes. GFAP positive staining showed green fluorescence primarily located in the cytoplasm and synapses of astrocytes [21]. DAPI was specifically located in the nuclei of the astrocytes (Fig. 3D) [22]. The somas of astrocytes were small, and the projections were elongated and radial in the CG (Fig. 3D). Compared with those of the CG, the processes of the immune-positive astrocytes were thickened, extended, dense, and interlaced in the SG-0h (Fig. 3D). We measured the PL and PMD of the astrocytes in the immunofluorescent images using Imaris-x64 9.2.1 software (Bitplane, Zurich, Switzerland). Compared with the CG, the greatest changes in the PL (P < 0.001) and PMD (P < 0.01) were observed at 0 h post stimulation (SG-0h, i.e., immediately after stimulation) (Fig. 3A-B). The morphological changes in astrocytes recovered to their baseline state at 1 h post-stimulation (SG-1h, P > 0.05) (Fig. 3A-B).

The immunoreactivity of GFAP was also increased, an indication that its expression was upregulated. Image-pro plus 6.0 (Media Cybernetics Inc., Rockville, MD, USA) and AlphaEase FC (Alpha Innotech, San Leandro, CA, USA) soft were used to obtain the MOD values of immunofluorescence (Fig. 3C). Compared with the CG, astrocytes also exhibited an increase in the expression of GFAP following neuronal excitation (SG-0h, P < 0.01) (Fig. 3C). The results of the western blot analysis showed a significant increase in the ratios of the grayscale values of the target bands to those of the internal reference bands in SG-0h compared to the CG (P < 0.01) (Fig. 3E-F). Hence, morphological changes in astrocytes and expression of GFAP induced by electrical stimulation were most obviously changed at the initial timepoint after stimulation (SG-0h), and these aforementioned changes in astrocytes were gradually restored within 1 hour (SG-1h) (Fig. 3).

**Figure 5.**
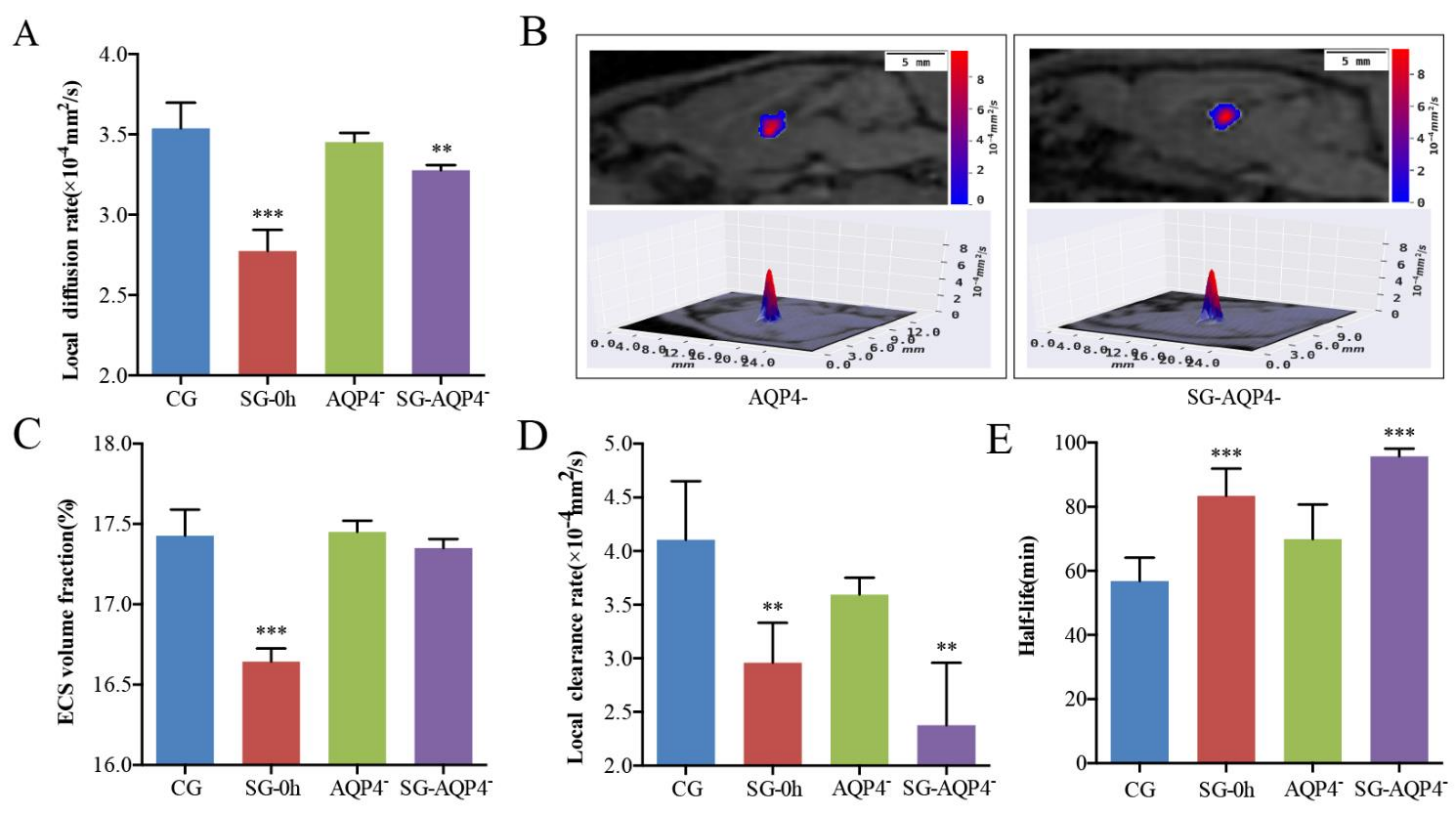
Effects of electrical stimulation on the diffusion parameters of the ECS structure and ISF drainage in the thalamus of *AQP4* knockout rats. (A, C-E) Tracer-based MRI showed that the biophysical parameters of the ECS structure and ISF drainage in the thalamus of the *AQP4* knockout rats were similar to those in the thalamus of the CG. Meanwhile, electrical stimulation did not result in significant changes in the α of the thalamus. However, electrical stimulation after *AQP4* knockout significantly reduced the D_ECS_ and ISF drainage in the thalamus and significantly increased the half-life of ISF, similar to the stimulation group. (B) The contour maps of the D_ECS_-mapping images showed that compared with the *AQP4* deficiency group, no significant differences were observed in the patterns and amplitudes of the contour maps of D_ECS_ in the *AQP4* deficiency with electrical stimulation group (SG-*AQP4*^-^) rats. Data are expressed as the mean ± standard deviation. ** P < 0.01, *** P < 0.001. Abbreviations: ECS: extracellular space; ISF: interstitial fluid; CG: control group; SG: stimulation group; *AQP4*^-^: *AQP4*^-^deficient group; SG-*AQP4*^-^: stimulation and *AQP4* deficiency group; D_ECS_: effective diffusion coefficient of the ECS; α: volume fraction.

**Figure 6.**
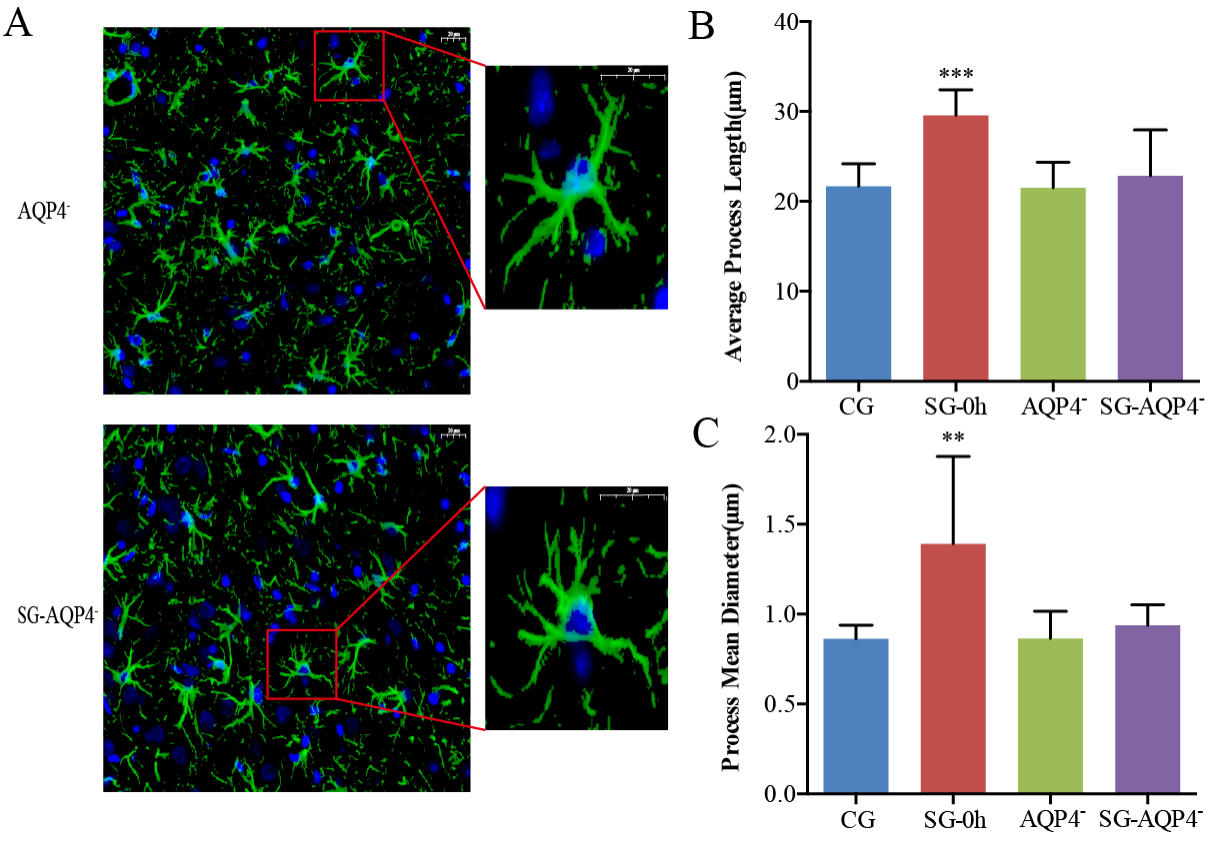
Effects of electrical stimulation on the morphological changes in the thalamus of *AQP4* knockout rats. (A-C) Astrocytes were specifically labelled with immunofluorescence, and the PL and PMD of the astrocytes were analyzed. Compared with the CG, the PL and PMD of the astrocytes were not changed in the *AQP4*^-^ and SG-*AQP4*^-^ groups. Data are expressed as the mean ± standard deviation. ** P < 0.01, *** P < 0.001. Abbreviations: CG: control group; SG: stimulation group; *AQP4*^-^: *AQP4*^-^deficient group; SG-*AQP4*^-^: stimulation and *AQP4* deficiency group; PL: process length; PMD: process mean diameter.

### Changes in the metabolism of neurotransmitters in the ECS after pain stimulation

We also investigated the biochemical mechanisms underlying the electrical stimulation-induced changes in ECS diffusion parameters in the thalamus. We used MSI to detect the relative changes of metabolites in the ECS during stimulation process. The results of the MSI showed that external electrical stimulation affected many metabolized substances in the rat thalamus. These substances were related to antioxidants, the glutamate-glutamine cycle, the malate-aspartate shuttle, and balance of inorganic ions (Fig. 4).

Compared with the CG, electrical stimulation caused changes in intracellular and extracellular Na^+^ and K^+^ ions in the SGs (Fig. 4A, E). The peak value post electrical stimulation was at 1.5 h for Na^+^ (P < 0.001), and 2 h for K^+^ (P < 0.001) (Fig. 4A, E). External electrical stimulation increased the amount of antioxidants in the rat thalamus, such as glutathione (P < 0.001), ascorbic acid (P < 0.001), and taurine (P < 0.01) in the SG-2h group (Fig. 4B-D, F). This stimulation also enhanced the glutamate-glutamine cycle and malate-aspartate shuttle in the rat thalamus and effected dynamic changes in excitatory neurotransmitters, such as glutamate, glutamine, aspartate, and N-acetylaspartate (Fig. 4G-K). The greatest changes in the concentrations of these neurotransmitters were observed in the SG-2h group (P < 0.001). The neurotransmitters levels were effectively restored after 3 h (SG-3h, P > 0.05) (Fig. 4G-K).

### Effects of pain stimulation on ECS diffusion properties in the thalamus of AQP4 knockout rats

To observe the effects of the *AQP4* on the ECS structure and ISF drainage parameters of the ECS in the thalamus, we performed tracer-based MRI and *in vivo* D_ECS_-mapping in the rats of *AQP4*^-^ and SG-*AQP4*^-^ groups (Fig. 5). Surprisingly, *AQP4* knockout did not induce changes in the D_ECS_ and α of the *AQP4*^-^ rats as compared with the CG (P > 0.05) (Fig. 5A-C). Meanwhile, electrical stimulation did not result in significant changes in the α of the thalamus in the SG-*AQP4*^-^ rats (P > 0.05) (Fig. 5C). However, electrical stimulation caused a significant decline in the D_ECS_ of the thalamus in the SG-*AQP4*^-^ rats (P < 0.05) (Fig. 5A). In addition, to obtain the structural parameters of the ECS, we also monitored the effect of electrical stimulation on ISF drainage in the thalamus of the *AQP4*^-^ rats. *AQP4* deletion did not affect the k’ and t_1/2_ in the ECS of the thalamus (Fig. 5D-E). However, electrical stimulation led to a decrease in the k’ (P < 0.01), while an increase in the t_1/2_ (P < 0.001) of ISF in the thalamus of SG-*AQP4*^-^ rats (Fig. 5D-E).

**Figure 7.**
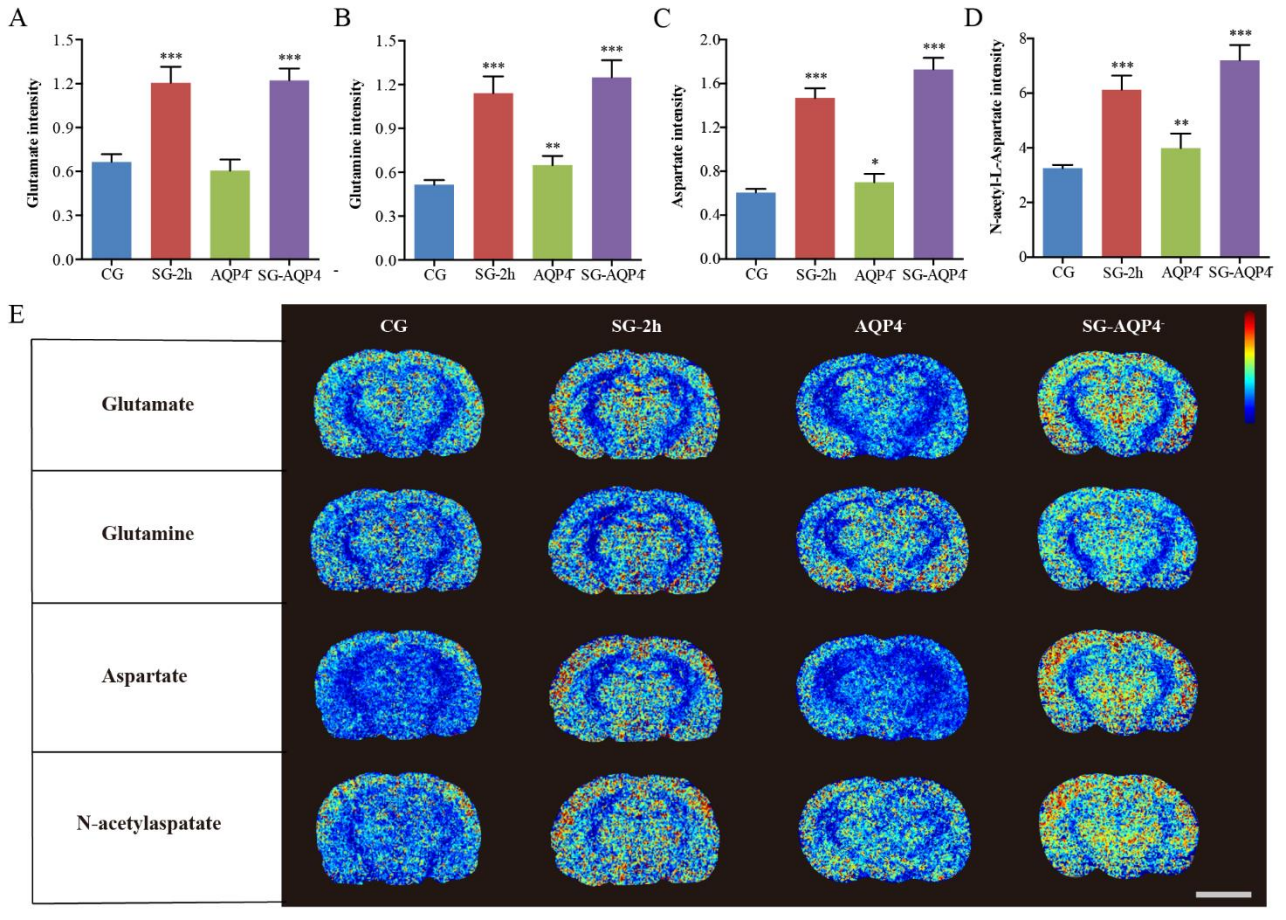
Effects of electrical stimulation on the biochemical changes in the thalamus of *AQP4* knockout rats. (A-E) Compared with the CG, external electrical stimulation also equally increased excitatory neurotransmitters amounts, such as glutamate, glutamine, aspartate, and N-acetylaspartate in the *AQP4* knockout rats. (H) The results from the MSI images showed that the average density of metabolites significantly increased after electrical stimulation compared with the average density of metabolites in the CG (scale bar, 5 mm). Data are expressed as the mean ± standard deviation. * P < 0.05, ** P < 0.01, *** P < 0.001. Abbreviations: CG: control group; SG: stimulation group; *AQP4*^-^: *AQP4*^-^deficient group; SG-*AQP4*^-^: stimulation and *AQP4* deficiency group; PL: process length; PMD: process mean diameter; MSI: mass spectrometry imaging.

Image processing software was used to analyze the average PL and PMD of astrocytes in immune-fluorescence images of *AQP4* knockout rats. The results showed that morphological changes in astrocytes following electrical stimulation were not found in the *AQP4*^-^ and SG-*AQP4*^-^ groups, which further verified the results obtained by tracer-based MRI (Fig. 6A-C). Especially, electrical stimulation did not result in significant changes in the α of the thalamus in the SG-*AQP4*^-^ rats compared with the CG (Fig. 6A-C). Based on the fact that electrical stimulation did not cause changes in the ECS structural parameters of *AQP4* knockout rats, we further performed MSI to study the biochemical mechanisms in the thalamus of *AQP4* knockout rats after 2 hours following electrical stimulation. Compared with the CG, the MSI results showed that external electrical stimulation equally increased excitatory neurotransmitters, such as glutamate, glutamine, aspartate, and N-acetylaspartate in the *AQP4* gene knockout rats (Fig. 7A-E). The aforementioned further validates our hypothesis that the downregulation of ISF drainage following neuronal excitation is mainly attributed to the biochemical changes rather than the morphological changes in the brain ECS.

### Diffusion in the brain ECS can track neurotransmitters release following neuronal excitation

**Figure 8.**
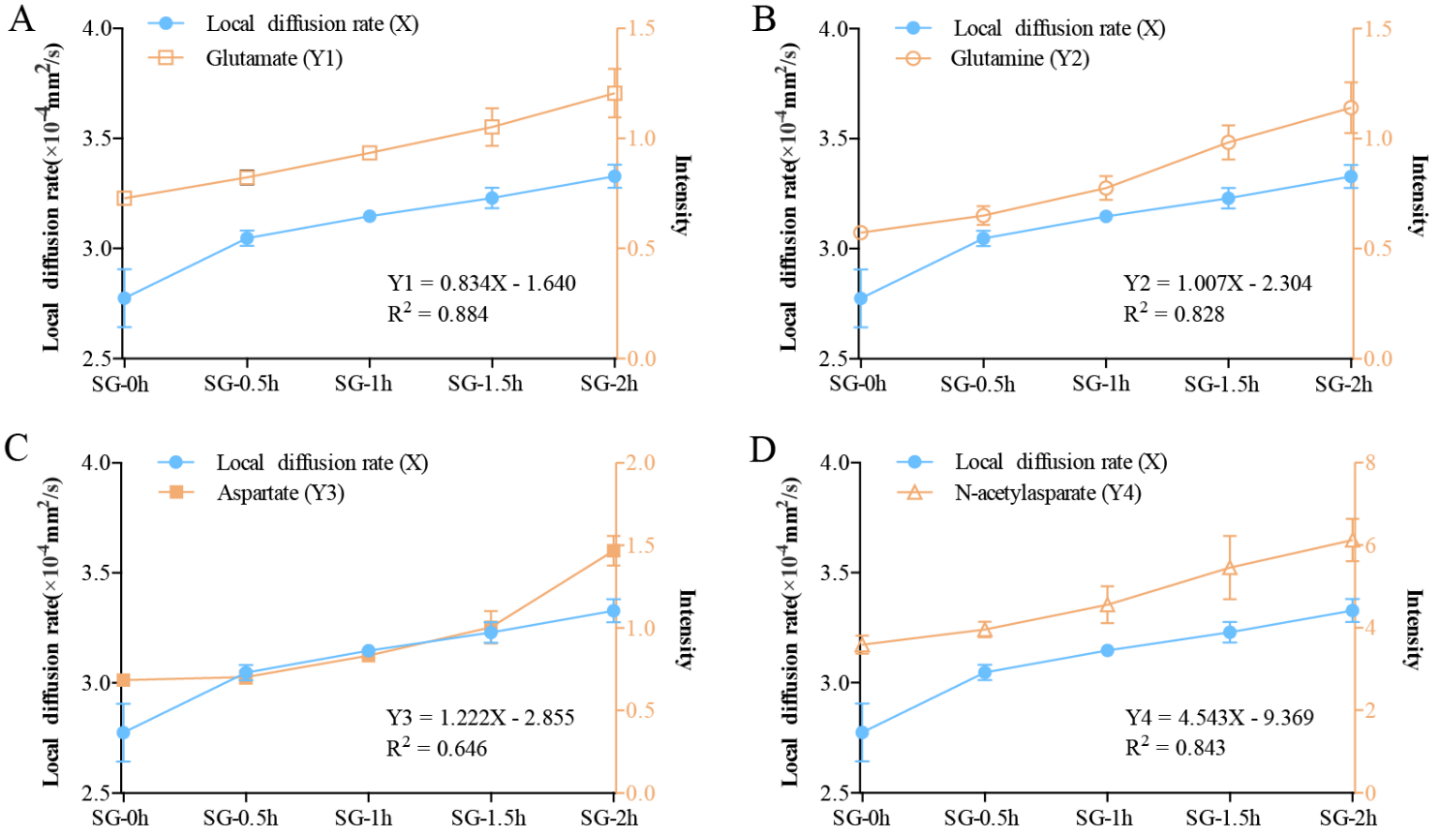
Linear regression analysis between the D_ECS_ obtained from the dynamic detection within 2 h after neuronal excitation and the dynamic changes in the metabolism of substances after excitation obtained from biological mass spectrometry. (A, B, D) There was a significant linear relationship between the dynamic changes in the D_ECS_ within 2 h of neuronal excitation and the detected dynamic changes in the metabolism of substances, such as glutamate, glutamine, and N-acetylaspartate. (C) The linear relationship between the dynamic changes of the D_ECS_ and the detected dynamic changes in aspartate was not significant. Data are expressed as the mean ± standard deviation. * P < 0.05. Abbreviations: CG: control group; SG: stimulation group; D_ECS_: effective diffusion coefficient of the ECS.

To assess whether the optimized tracer-based MRI method specifically monitors neuronal excitation-induced changes in the metabolic activities of neurons in the living brain, we conducted a linear regression analysis between the D_ECS_ obtained from the dynamic detection within 2 h after neuronal excitation and the dynamic changes in the metabolism of substances after excitation as obtained from the MSI (Fig. 8). The results showed that there was a statistically significant linear relationship between the dynamic changes in the D_ECS_ and the detected dynamic changes in the metabolism of substances, e.g., linear relationships were seen for glutamate (Y_1_ = 0.834X-1.640, R^2^ = 0.884, Sig = 0.017 < 0.05), glutamine (Y_2_ = 1.007X-2.304, R^2^ = 0.828, Sig = 0.032 < 0.05) and N-acetylaspartate (Y_4_ = 4.543X-9.369, R^2^ = 0.843, Sig = 0.028 < 0.05) (Fig. 8A-B, D). However, the linear relationship between the dynamic changes in D_ECS_ and the detected dynamic changes in aspartate levels (Y_3_ = 1.222X-2.855, R^2^ = 0.646, Sig = 0.101 > 0.05) was not significant (Fig. 8C). These results showed that the diffusion coefficient of ECS based on the linear regression correlation parameters could improve the accuracy of predicting metabolic changes (especially glutamate) induced by neuronal excitation.

## DISCUSSION

Substance transportation in the brain ECS is a complex process involving numerous factors. Various physiological and pathological variations can alter ECS structure and ISF drainage [1, 3, 6, 23, 24]. Previous studies have shown that under the condition of transient or long-lasting neuronal excitation, the morphological changes of astrocytes *ex vivo* in the cortex can reduce the volume fraction of ECS by approximately 5-30% [25-30]. Based on the fact that astrocytes play an active role in regulating structural changes in state-dependent network activity. Therefore, to assess the morphological basis of the ECS structural parameters in the thalamus caused by external electrical stimulation, astrocytes were used as our primary target. Our data showed that activated astrocytes play a role in the initiation of acute pain, accompanied by change in GFAP expression. We observed that GFAP staining was rapidly expressed within as little as several minutes, even 0 hour; our data were similar to this previous report [31]. However, what we need to explain this result at 0 hour was that it took a few minutes from electrical stimulation to collecting brain samples. In the present study, using an advanced detection principle and innovative technology of tracer-based MRI, a 4% reduction of ECS volume fraction and a significant slowdown of ISF drainage were shown at the thalamus following the painful stimulation, which was a more objective reflection of the changes in the brain ECS parameters *in vivo* (Fig. 2H).

Two potential mechanisms have previously been proposed to explain the down-modulation of brain ISF following neuronal excitation [11], including a biomechanical mechanism related to the structural change in astrocytes and a biochemical mechanism involving the release of neurotransmitters, both of which could impede molecular motion in the brain ECS. The main aquaporin isoform expressed on the astrocyte foot processes in the brain parenchyma is *AQP4*. In an excitatory state, *AQP4* is implicated in the metabolic transport of water in glial cells, thereby regulating the volume of the brain ECS. Notably, our results showed that the morphological changes in the astrocytes were suppressed in the *APQ4* knockout model following neuronal excitation, while the slowdown of D_ECS_ and ISF drainage was the same as that in the SG (Fig. 5A, C) [32, 33]. The results indicated that the downregulation of ISF drainage following neuronal excitation is mainly attributed to the biochemical changes rather than to morphological changes in the brain ECS.

Specifically, when neurons are excited, the active transmembrane water exchange driven by the ionic pump is directly coupled to their metabolic activity, thereby maintaining the balance of Na^+^ and K^+^ across the neuronal and glial cell membranes [34]. Notably, an opposite change in Na^+^/K^+^ levels in the excited and no net change in the amount in the brain is a classical principle of neurophysiology [34]. However, in this study, we observed that there was a slight elevation in Na^+^ levels at 1.5 h associated with a marked increase in K^+^ levels at 2 h in the brains after electrical stimulation (Fig. 4). Under physiological conditions, action-potential generation is accompanied by Na^+^influx and K^+^ outflow in the neurons; however, under some pathological conditions, both Na^+^and K^+^ concentrations have been found to be markedly elevated in the cortex of an acute ischemia rat model [35], brain injury model [36], and especially, in a pain rat model [37]. This phenomenon was possibly because the damaged neurons release marked amounts of glutamate and this excitatory transmitter stimulates Na^+^and K^+^excretion from the astrocytes. Glutathione, ascorbic acid, and taurine are important antioxidants in the body, and external stimulation increases their level to combat oxidative stress. This indicates the safety and controllability of our selected source of external stimulation on the regulation of substances in the brain ECS [38]. Neuronal excitation is accompanied by the release of various excitatory neurotransmitters into the brain ECS. In the current study, by using MSI, we found that during excitation, glutamate, glutamine, N-acetylaspartate and aspartate are released, which play a crucial role in maintaining neuronal excitability and inter-neuronal crosstalk [28, 39, 40]. The transient changes in the chemical components in this small space might lead to a series of changes in the local biochemical properties, such as colloid osmotic pressure, pH, and local charges [41]. Such factors, as well as the physical surface tension and siphon effect are all likely to impede molecular diffusion within the ECS [42, 43]. In the present study, the dynamic changes in D_ECS_ within the ECS division of the thalamus was synchronized with the release processes of excitatory neurotransmitters, indicating that the down-regulation of ISF drainage primarily depends on the diffusion decline caused by the release of neurotransmitters (Fig. 8). Importantly, this observed phenomenon could potentially form the basis for novel imaging techniques that dynamically track neuronal activities. The relative inhibitory weights of the factors that affect diffusion should be evaluated further in the future to verify our hypothesis and to confirm its potential for imaging use [44].

Functional MRI (fMRI) and positron emission tomography (PET) are the most popular imaging methods for detecting neuronal activities in the living brain [45, 46]. The weakly increased signal in the cortex on fMRI after neuronal activity was considered to be mainly caused by the oxidation process in the vascular compartment [46]. However, after inhibiting the signal contribution of the neurovascular coupling response, the slow water diffusion in the cortex has been shown to be more sensitive to neuronal activities [47, 48]. With the help of radioactively labelled precursors, PET can be used to visualize the action of the neurotransmitters in the brain [49, 50]. Diffusion fMRI represents a promising technique to reflect neuronal activities, but this method cannot differentiate among all water molecules on the voxel level of the cortex, including blood vessels, cells, and ECS. Notably, whether the modulation of diffusion originates from the intracellular or extracellular environment remains unclear [51]. Because of the introduction of tracers that are specifically located within the ECS, our present results showed the same diffusion changes in the deep brain nucleus and further verified that the restricted diffusion partly originated from the decrease of diffusion within the ECS. In the future, based on the understanding of the diffusion properties of ECS obtained by the invasive methods, the establishment of non-invasive methods will become a reality.

In the present study, we clarified the regulatory mechanism of neuronal excitation on substance transportation in the brain ECS. Although the involvement of the neural network in the transmission and modulation of pain is well established [52, 53], the role of the ECS in pain perception and the formation of painful memory has not been fully characterized. Combining the previous findings with our present results, we propose the hypothesis that specific sensations and their memories are achieved through the joint work of the neural circuit and the ECS. Painful stimulation activates the neurons of the posterior lateral nucleus to release neurotransmitters in the ECS division of the thalamus. Because the ISF drains in a compartmentalized ECS system, the sensory-dependent neurotransmitters stay and act in the specific ECS division for approximately 2 h. During this stay, the produced lactic acid by glutamate astrocytic glycolysis has been verified to be associated with the formation of long-term memory [5, 54, 55]. The aforementioned local procedures are very similar to disk writing and recording in computer storage [56]. The recorded information might be copied, or mirror stored in the hippocampus by its physical connections with the thalamus [57, 58]. Our finding shows that the concentration of excitatory neurotransmitters persisted in the brain for a period of time, which potentially affords new insight into the regulation of pain memory. In conclusion, the downregulation of ISF drainage following neuronal excitation is caused by the restricted diffusion in the ECS, and D_ECS_ might be used to track the neuronal activities in the deep brain. Our findings provide new insights for understanding the processing of perceptual information in the brain and new mechanisms of neural networks- interacted with ECS after neuronal excitation.
